# Type 1 diabetes can present before the age of 6 months and is characterised by autoimmunity and rapid loss of beta cells

**DOI:** 10.1007/s00125-020-05276-4

**Published:** 2020-10-08

**Authors:** Matthew B. Johnson, Kashyap A. Patel, Elisa De Franco, William Hagopian, Michael Killian, Timothy J. McDonald, Timothy I. M. Tree, Clara Domingo-Vila, Michelle Hudson, Suzanne Hammersley, Rebecca Dobbs, EXE-T1D Consortium, Sian Ellard, Sarah E. Flanagan, Andrew T. Hattersley, Richard A. Oram

**Affiliations:** 1 Institute of Biomedical and Clinical Science, University of Exeter Medical School, Exeter, UK; 2 Pacific Northwest Research Institute, Seattle, WA USA; 3 Blood Sciences, Royal Devon & Exeter NHS Foundation Trust, Exeter, UK; 4 Department of Immunobiology, School of Immunobiology & Microbial Sciences, Kings College London, London, UK; 5 NIHR Biomedical Research Centre Guys and St Thomas’ NHS Foundation Trust and Kings College London, London, UK; 6 National Institute for Health Exeter Research Clinical Research Facility, Royal Devon & Exeter NHS Foundation Trust, Exeter, UK

**Keywords:** Autoimmunity, Genetic risk score, Neonatal diabetes, Type 1 diabetes

## Abstract

**Aims/hypothesis:**

Diabetes diagnosed at <6 months of age is usually monogenic. However, 10–15% of affected infants do not have a pathogenic variant in one of the 26 known neonatal diabetes genes. We characterised infants diagnosed at <6 months of age without a pathogenic variant to assess whether polygenic type 1 diabetes could arise at early ages.

**Methods:**

We studied 166 infants diagnosed with type 1 diabetes at <6 months of age in whom pathogenic variants in all 26 known genes had been excluded and compared them with infants with monogenic neonatal diabetes (*n* = 164) or children with type 1 diabetes diagnosed at 6–24 months of age (*n* = 152). We assessed the type 1 diabetes genetic risk score (T1D-GRS), islet autoantibodies, C-peptide and clinical features.

**Results:**

We found an excess of infants with high T1D-GRS: 38% (63/166) had a T1D-GRS >95th centile of healthy individuals, whereas 5% (8/166) would be expected if all were monogenic (*p* < 0.0001). Individuals with a high T1D-GRS had a similar rate of autoantibody positivity to that seen in individuals with type 1 diabetes diagnosed at 6–24 months of age (41% vs 58%, *p* = 0.2), and had markedly reduced C-peptide levels (median <3 pmol/l within 1 year of diagnosis), reflecting rapid loss of insulin secretion. These individuals also had reduced birthweights (median *z* score −0.89), which were lowest in those diagnosed with type 1 diabetes at <3 months of age (median *z* score −1.98).

**Conclusions/interpretation:**

We provide strong evidence that type 1 diabetes can present before the age of 6 months based on individuals with this extremely early-onset diabetes subtype having the classic features of childhood type 1 diabetes: high genetic risk, autoimmunity and rapid beta cell loss. The early-onset association with reduced birthweight raises the possibility that for some individuals there was reduced insulin secretion in utero. Comprehensive genetic testing for all neonatal diabetes genes remains essential for all individuals diagnosed with diabetes at <6 months of age.

**Figure:**
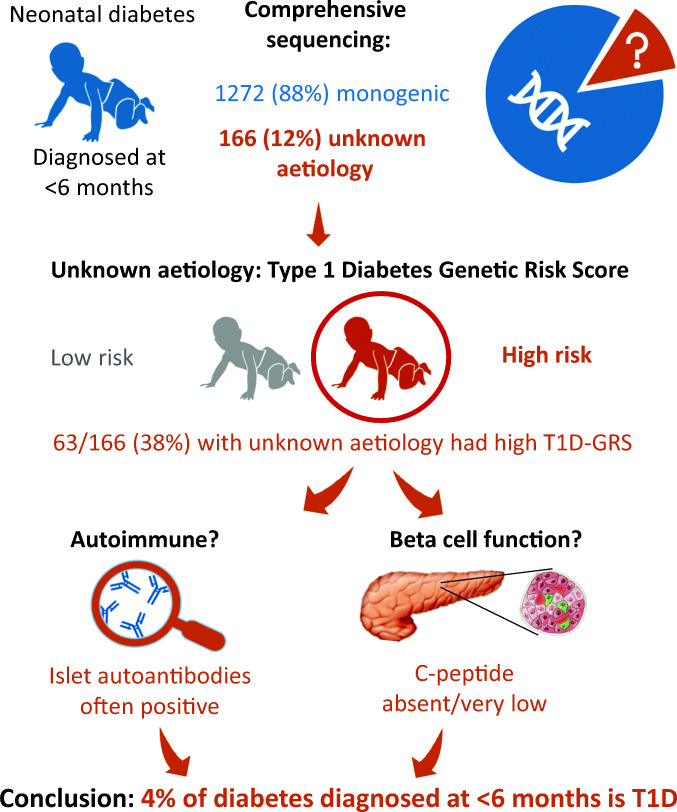
Graphical abstract

**Electronic supplementary material:**

The online version of this article (10.1007/s00125-020-05276-4) contains peer-reviewed but unedited supplementary material, which is available to authorised users.

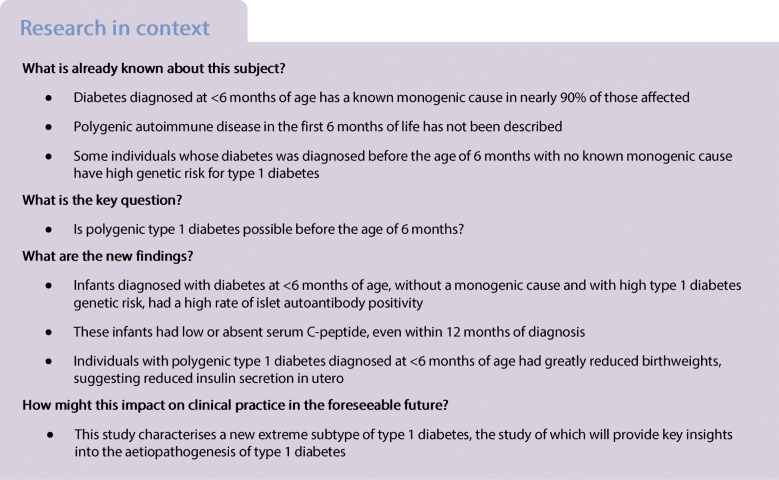



## Introduction

Diabetes that presents in the first 6 months of life (neonatal diabetes) has been thought to be exclusively caused by a pathogenic variant in a single gene; nearly 90% of individuals have one of 26 known causes depending on cohort definition, highlighting the need for comprehensive genetic testing [1–6]. The remaining ~10–15% may have a causative pathogenic variant in a gene or non-coding region that has not yet been identified. Another possibility is that they have polygenic type 1 diabetes and represent the extreme tail of the distribution of presenting age of type 1 diabetes [7].

Type 1 diabetes accounts for the vast majority of diabetes in childhood [8] and is a complex autoimmune condition caused by a combination of genetic risk and environmental factors. Established features of type 1 diabetes include positivity for islet-specific autoantibodies (GAD autoantibody [GADA], insulinoma antigen-2 autoantibody [IA2A] and zinc transporter 8 autoantibody [ZnT8A]) [9], and progression to low or absent C-peptide due to beta cell destruction and resultant insulin deficiency [10, 11]. There are rare examples of autoimmune diseases, including autoimmune diabetes, presenting before the age of 6 months but these are caused either by highly penetrant pathogenic variants in immune genes such as *FOXP3*, *STAT3* and *LRBA* [2, 3, 12] or, as for neonatal lupus, by passive transfer of pathogenic maternal antibodies [13]. To our knowledge, no polygenic autoimmune diseases (including type 1 diabetes) have been described and characterised in individuals below the age of 6 months.

We recently performed an analysis of type 1 diabetes genetic risk in people referred to our centre for genetic testing for monogenic diabetes [7]. We showed that a type 1 diabetes genetic risk score (T1D-GRS), which expresses genetic risk as a continuum, could be used to discriminate type 1 diabetes from monogenic diabetes including neonatal diabetes (NDM). Furthermore, we studied 48 infants diagnosed at age <6 months without a genetic pathogenic variant and identified an excess of infants with high polygenic type 1 diabetes risk, raising the hypothesis that there are children with polygenic type 1 diabetes diagnosed under the age of 6 months.

In this study, we aimed to characterise infants diagnosed with diabetes at <6 months of age without monogenic NDM who had high type 1 diabetes genetic risk, in order to confirm whether they have features consistent with type 1 diabetes and further define their phenotype.

## Methods

### Cohorts

We studied two groups of infants with diabetes onset at <6 months of age, and a group of children diagnosed with type 1 diabetes at older ages. The first group of infants had no detectable pathogenic variants in the 26 genetic loci known to cause NDM. The second group of infants had monogenic NDM with a detectable pathogenic variant.

#### Individuals with diabetes of unknown genetic aetiology diagnosed before 6 months of age

We studied 166/1438 (12%) individuals referred to our laboratory for genetic testing for permanent NDM between 2000 and 2019 in whom comprehensive targeted next-generation sequencing had excluded a pathogenic variant in the 26 known genes. Clinical information was provided by the referring physician via a referral form available at www.diabetesgenes.org.

#### Individuals with type 1 diabetes

We compared genetic risk for type 1 diabetes in our cohorts with a reference cohort of 1962 individuals with type 1 diabetes (from the Wellcome Trust Case Control Consortium [14]). We additionally compared their islet autoantibodies and C-peptide levels with those of 152 individuals diagnosed between 6 months and 2 years of age who were originally referred for monogenic diabetes testing but subsequently diagnosed with type 1 diabetes. We measured C-peptide (*n* = 64) and autoantibody levels (*n* = 88) in all available samples. Monogenic diabetes was excluded by comprehensive genetic testing in all individuals diagnosed at 6–9 months of age [15].

#### Individuals with monogenic diabetes diagnosed before 6 months of age

We used a comparator group of individuals with monogenic permanent NDM (*n* = 164) caused by either heterozygous pathogenic *ABCC8* (*n* = 25), *KCNJ11* (*n* = 72) or *INS* (*n* = 67) variants. We measured islet autoantibodies (*n* = 93) and serum C-peptide (*n* = 111), depending on sample availability.

### Genetic testing

#### Testing of the known genes

All individuals diagnosed in the first 9 months of life were tested by rapid Sanger sequencing of *ABCC8*, *KCNJ11* and *INS* and, if no pathogenic variant was identified, via targeted next-generation sequencing (tNGS) of all 26 known genetic causes of permanent NDM (electronic supplementary material [ESM] Table 1). Historic samples were tested for new genetic causes as they were discovered. This assay can detect single nucleotide variants, insertion–deletions, copy number variants and structural variation [16].

#### T1D-GRS

We generated the T1D-GRS as previously described [17]. Briefly, we genotyped single SNPs tagging the top 30 risk alleles for type 1 diabetes (ESM Table 2) and summed their log_10_-transformed ORs before dividing by the total number of alleles to obtain a numeric score. High/low T1D-GRS was defined as an individual’s score being above or below 0.280, the 95th centile of 4862 control individuals without type 1 diabetes [14].

#### Biomarker measurement

EDTA–blood samples were collected to extract DNA for genetic testing. All samples were spun at 1300 *g* for 8 min and, where it could be separated, plasma was stored at −80°C.

#### Antibody measurement

Antibody testing was undertaken by radiolabel assays at the Pacific Northwest Diabetes Research Institute, USA. Cut-offs for positivity were defined as the 99th percentile of >200 individuals from a control healthy population [18]. The laboratory participates in the Diabetes Autoantibody Standardization Program (DASP)/Islet Autoantibody Standardization Program (IASP) proficiency testing and each separate autoantibody assay is Clinical Laboratory Improvement Amendments (CLIA) certified (CLIA no. 50D0982418).

#### C-peptide measurement

C-peptide was measured in EDTA–plasma from samples of blood couriered or posted to the laboratory primarily for genetic testing. These samples were usually postprandial but the relationship to the eating of meals was not stipulated. We have previously demonstrated the stability of C-peptide in EDTA–plasma [19]. C-peptide was analysed using a direct electrochemiluminescence immunoassay (Roche Diagnostics, Mannheim, Germany) as previously described [20]. The limit of detection of the assay is 3 pmol/l and for statistical analysis we coded values below the limit of detection as 2.9 pmol/l.

### Ethics approval

All study participants gave informed consent or assent was obtained where children were too young and parental consent was provided, in accordance with the declaration of Helsinki. This study was approved by the Genetic Beta Cell Research Bank, Exeter, UK. Ethical approval was provided by the North Wales Research Ethics Committee, UK (IRAS project ID 231760).

### Statistical analysis

The Mann–Whitney *U* and Kruskal–Wallis tests were used to compare continuous variables and the Fisher’s exact test was used to compare categorical variables. Statistical analysis was undertaken in Stata14 (StataCorp, College Station, TX, USA). Birthweight *z* scores (adjusted for sex and gestational age) were calculated based on WHO international reference ranges [21]. We performed sensitivity analyses stratified by ethnicity. A *p* value of <0.05 was used to define statistical significance.

## Results

### High T1D-GRS identifies probable type 1 diabetes diagnosed before the age of 6 months

**Fig. 1:**
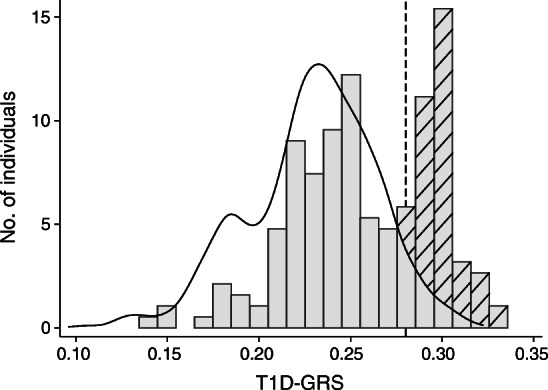
Distribution of T1D-GRS in control population (individuals without diabetes) (black line, *n* = 2938) and individuals with diabetes diagnosed at <6 months of age without a known genetic cause (grey bars, *n* = 166). Hatched bars represent the enrichment of individuals with high T1D-GRS, above the control population distribution. The dashed line represents the 95th centile of the control population (0.280)

We identified an excess of infants with high T1D-GRS in the group diagnosed before the age of 6 months without a monogenic diagnosis (Fig. 1). Of the 166 infants, 63 (38%) had a T1D-GRS above the 95th centile of the healthy control children without type 1 diabetes (>0.280; Table 1). Only 8 (5%) would be expected to have scores this high if these individuals all had a novel undiscovered monogenic aetiology (*p* < 0.0001), as individuals with monogenic diabetes have the same distribution of T1D-GRS as seen in individuals without type 1 diabetes (i.e. 5% above the 95th centile). The excess of individuals with high T1D-GRS suggests that this subset of individuals has polygenic type 1 diabetes. This also suggests that type 1 diabetes accounts for at least 4% (excess no. of individuals [observed−expected]/total no. of individuals; [63–8]/1438) of our international cohort of 1438 individuals from 108 countries who were diagnosed with diabetes before the age of 6 months.

**Table 1:** Clinical and demographic features of individuals with type 1 diabetes diagnosed in the first 6 months of life

Characteristic	Monogenic NDM (*n* = 164)	Diabetes diagnosed before 6 months of age		Type 1 diabetes diagnosed at 6–24 months of age (*n* = 152)
		Low T1D-GRS (*n* = 103)	High T1D-GRS (*n* = 63)	
T1D-GRS	0.233 (0.217, 0.245) (*n* = 115)	0.241 (0.219, 0.252)	0.298 (0.292, 0.304)	0.294 (0.261, 0.310) (*n* = 102)
Age at diagnosis (weeks)	9 (4, 15)	4 (1, 12)	16 (6, 21)	48 (39, 56)
Birthweight *z* score^a^	−1.39 (−2.04, −0.62) (*n* = 124)	−1.23 (−2.54, −0.17) (*n* = 81)	−0.89 (−1.96, −0.02) (*n* = 48)	−0.25 (−0.89, 0.59) (*n* = 125)
Female sex, *n* (%)	86 (52)	40 (39)	27 (43)	60 (40)
Related parents, *n*/*n* (%)^b^	20/157 (13)	24/100 (24)	15/63 (24)	12/89 (13)
First-degree relative with type 1 diabetes, *n*/*n* (%)	1/152 (1)	1/97 (1)	10/61 (16)	11/88 (13)
Mother with type 1 diabetes, *n*/*n* (%)	0/152 (0)	0/97 (0)	1/61 (2)	2/88 (2)
Father with type 1 diabetes, *n*/*n* (%)	1/152 (1)	0/97 (0)	4/61 (7)	3/88 (3)
Sibling with type 1 diabetes, *n*/*n* (%)	0/152 (0)	1/97 (1)	6/61 (10)	6/88 (7)
Insulin treated from diagnosis, *n*/*n* (%)	164/164 (100)	103/103 (100)	63/63 (100)	152/152 (100)
Insulin daily dose, U/kg	0.77 (0.50, 1.00) (*n* = 125)	0.63 (0.50, 1.00) (*n* = 81)	0.81 (0.50, 1.00) (*n* = 48)	0.78 (0.50, 1.00) (*n* = 64)
HbA_1c_, mmol/mol	69 (56, 101) (*n* = 107)	64 (44, 74) (*n* = 49)	65 (58, 78) (*n* = 54)	76 (62, 87) (*n* = 59)
HbA_1c_, %	8.5 (7.3, 11.4)	8.0 (6.2, 8.9)	8.1 (7.5, 9.3)	9.1 (7.8, 10.1)
Blood glucose at diagnosis, mmol/l	30.7 (22.0, 38.9) (*n* = 114)	28.0 (21.1, 39.2) (*n* = 80)	30.0 (23.0, 38.1) (*n* = 52)	27.7 (23.0, 36.1) (*n* = 59)
Duration of diabetes at sampling	5.5 m (1.0 m, 8.2 y)	1.5 m (0.7 m, 1.7 y)	7.9 m (2.2 m, 5.3 y)	2.9 y (1.5 m, 15.1 y)
Syndromic presentation, *n* (%)	5 (3)	28 (27)	6 (9)	1 (1)
Additional autoimmune condition, *n* (%)	1 (1)	7 (7)	3 (4)	15 (10)
Autoantibody positive, *n*/*n* (%)				
GADA/IA2A/ZnT8A	7/93 (8)	4/33 (12)	9/22 (41)	51/88 (58)
GADA	2/93 (2)	4/33 (12)	6/22 (27)	39/88 (44)
IA2A	4/93 (4)	0/33 (0)	4/22 (16)	13/88 (15)
ZnT8A	1/93 (1)	0/33 (0)	3/22 (14)	7/88 (8)
Age at antibody measurement	26 w (13 w, 5.8 y)	20 w (5 w, 9 y)	3.7 y (4.6 m, 8.5 y)	4 y (1 y, 17 y)
Duration of diabetes at antibody measurement	11 w (3 w, 6 y)	6 w (3 w, 9 y)	3.0 y (2.3 m, 7.5 y)	4.0 y (6 w, 14 y)
C-peptide, pmol/l				
Duration of diabetes <12 months	64 (13, 138) (*n* = 61)	117 (16, 362) (*n* = 15)	<3 (<3, 10) (*n* = 7)	24.5 (<3, 67) (*n* = 26)
Duration of diabetes >12 months	8.5 (<3, 40) (*n* = 50)	<3 (<3, 63) (*n* = 10)	<3 (<3, <3) (*n* = 12)	<3 (<3, <3) (*n* = 38)

All data are median (IQR) unless otherwise specified

^a^Based on WHO international reference range, adjusted for sex and gestation period

^b^Defined as parents being second cousins or closer relatives

m, months; w, weeks; y, years

Performing the analysis only on white Europeans (*n* = 45) identified a similar proportion of individuals with a T1D-GRS above the 95th centile of healthy control individuals (18/45 [40%] vs 63/166 [38%], *p* = 0.9). This suggests that the results are not due to the population structure of our cohort. We therefore defined the 63 children with scores above the 95th centile as having likely type 1 diabetes and hypothesised that they would have features of polygenic type 1 diabetes.

### Islet autoantibody prevalence in children diagnosed before the age of 6 months with a high T1D-GRS

**Fig. 2:**
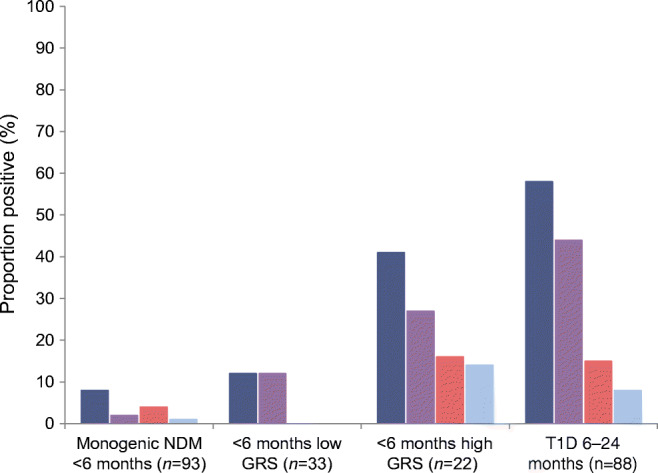
Proportion of infants positive for anti-islet autoantibodies in those with diabetes of a known monogenic cause (*n* = 93, control group), diabetes with an unknown cause diagnosed before the age of 6 months with a low T1D-GRS (*n* = 33) and with a high T1D-GRS (*n* = 22), and type 1 diabetes diagnosed between the ages of 6 months and 2 years (*n* = 88). Dark blue bars, positive for at least one of GADA, IA2A or ZnT8A; purple bars, GADA positive; red bars, IA2A positive; light blue bars, ZnT8A positive. GRS, genetic risk score; T1D, type 1 diabetes

Infants diagnosed with diabetes before the age of 6 months with a high T1D-GRS and no known cause had islet-specific autoantibody prevalence similar to that found in children with type 1 diabetes diagnosed between 6 months and 2 years of age. Serum was available for autoantibody measurement from 22/63 of the children with a high T1D-GRS and no known cause. Of these 22 children, nine (41%) were positive for at least one of GADA, IA2A or ZnT8A (Fig. 2). This was similar to the proportion of autoantibody-positive individuals with type 1 diabetes diagnosed between 6 months and 2 years of age (9/22 vs 51/88, *p* = 0.2) (Fig. 2) who had similar duration of diabetes to those with diabetes diagnosed at <6 months of age with a high T1D-GRS (median duration 3.0 vs 4.0 years, *p* = 0.3) (Table 1). The proportion of infants who were diagnosed with diabetes at <6 months of age and who had a high T1D-GRS that were positive for an antibody was higher than that of infants with confirmed non-autoimmune monogenic diabetes (9/22 vs 7/93, *p* = 0.0004). Of the infants who were diagnosed with diabetes at <6 months of age and who had a high T1D-GRS, 2/22 (10%) were positive for two antibodies and one was positive for all three antibodies. The characteristics of those where antibody testing was possible vs not possible were similar (ESM Table 3).

As all infants were insulin treated at referral, we were unable to discern insulin autoantibody (IAA) status pertaining to endogenous insulin from IAAs to exogenous insulin. That notwithstanding, we found a higher proportion of infants positive for IAAs in those with likely type 1 diabetes diagnosed at <6 months of age than in those with monogenic NDM (18/22 vs 38/98, *p* = 0.0003) or those with a low T1D-GRS (18/22 vs 12/28, *p* = 0.008) (ESM Fig. 1). This was particularly the case if the duration of insulin treatment was ≤3 months (4/8 vs 6/75, *p* = 0.006) (ESM Fig. 2). However, as duration of insulin treatment increased, the proportion in both groups was similar, presumably due to accumulation of antibodies to exogenous insulin with prolonged exposure.

### Evidence for severe insulin deficiency

**Fig. 3:**
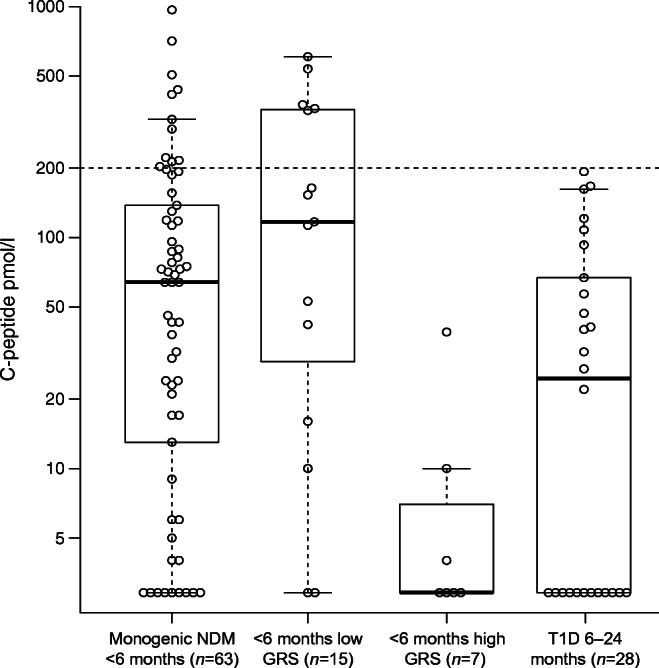
Serum C-peptide (pmol/l) in infants with diabetes of a known monogenic cause (*n* = 63, control group), diabetes with an unknown cause diagnosed before the age of 6 months with a low T1D-GRS (*n* = 15) and with a high T1D-GRS (*n* = 7), and type 1 diabetes diagnosed between the ages of 6 months and 2 years (*n* = 28). C-peptide is plotted on a log scale. The dashed horizontal line represents 200 pmol/l, with C-peptide values below this considered low. All samples were taken within 1 year of the diagnosis of diabetes. The central line within the box represents the median and the upper and lower limits of the box represent the IQR. The whiskers are the most extreme values within 1.5× the IQR from the first and second quartiles. GRS, genetic risk score

We found that the high T1D-GRS group had low C-peptide levels at the time of referral for genetic analysis for all durations of diabetes. Within 12 months of diabetes diagnosis (median duration 1 month [IQR 0.3–3], *n* = 7), the children with diabetes diagnosed before the age of 6 months and a high T1D-GRS had lower C-peptide levels than the infants with monogenic diabetes (median <3 vs 64 pmol/l, *p* = 0.003) and those with diabetes diagnosed before the age of 6 months and a low T1D-GRS (<3 vs 117 pmol/l, *p* = 0.004) (Fig. 3). The high T1D-GRS group diagnosed before the age of 6 months had similar C-peptide levels to the group diagnosed with type 1 diabetes between the ages of 6 months and 2 years (<3 vs 24.5 pmol/l, *p* = 0.22). The same pattern was observed when including all diabetes durations (ranging from 2 days to 20 years) (ESM Fig. 3).

In infants with diabetes diagnosed before the age of 6 months, all three biomarkers of type 1 diabetes (high T1D-GRS, positive for an islet autoantibody, low/absent C-peptide) were present in six from 19 tested for all three.

### Low birthweight is a characteristic of very-early-onset diabetes

**Fig. 4:**
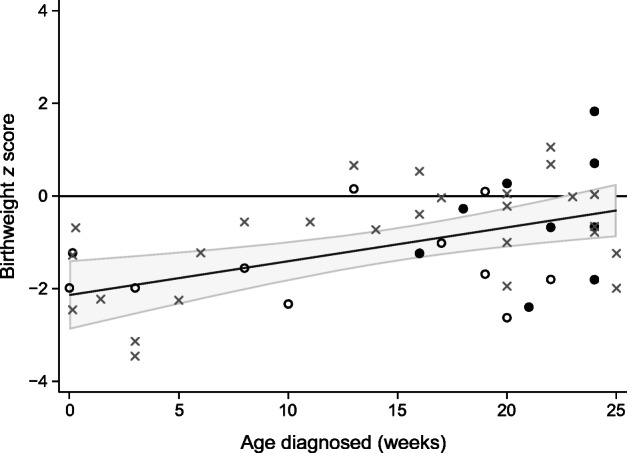
Scatter plot of adjusted birthweight *z* score and age at diagnosis of diabetes in weeks in children with high T1D-GRS (*n* = 48). The fitted line is the predicted linear regression (*r*^2^ = 0.18, *p* = 0.001) with grey shading representing the 95% CI. Black circles, islet autoantibody positive; white circles, islet autoantibody tested; grey crosses, islet autoantibody testing unavailable

We found that the infants who were diagnosed with diabetes at <6 months of age with high T1D-GRS had reduced birthweight compared with the WHO international reference population (median *z* score −0.89 [IQR −1.96, −0.02], *n* = 48) [21]. Interestingly, we found earlier age at diagnosis of diabetes was associated with lower birthweight (*r*^2^ = 0.18, *p* = 0.001) (Fig. 4), with individuals diagnosed in the first 3 months of life having markedly reduced birthweight (*z* score: −1.98 [IQR −2.33, −1.23], *n* = 17). These individuals with the earliest onset (<3 months) had similar T1D-GRS (0.299 vs 0.296, *p* = 0.3) and C-peptide values (2.9 vs 2.9, *p* = 0.4) to those diagnosed at 3–6 months of age (ESM Table 4). In those with the earliest onset, 3/7 were positive for IAA; however, none were positive for GADA, IA2A or ZnT8A, possibly due to the extremely young age of onset in these individuals. Children diagnosed with type 1 diabetes between the ages of 6 months and 2 years had birthweights similar to those of the normal population (*z* score −0.25 [IQR −0.89, 0.59], *n* = 125, Table 1), suggesting that reduced birthweight is not a feature in later-onset type 1 diabetes. Infants with monogenic NDM also had reduced birthweights (median −1.39), as genetic beta cell defects reduce insulin secretion and therefore insulin-mediated growth in utero.

There was also a relationship between lower birthweight and earlier diagnosis for individuals diagnosed before the age of 6 months with low GRS (ESM Fig. 4). This likely reflects reduced insulin secretion in utero as seen in known forms of monogenic NDM.

### Clinical and demographic features of type 1 diabetes diagnosed at <6 months

We analysed the clinical and demographic information for individuals with type 1 diabetes diagnosed at <6 months of age defined by high T1D-GRS (Table 1). The median age of diabetes diagnosis of these individuals was 16 weeks (range 1–25 weeks; IQR 6, 21 weeks). The youngest individual with a positive islet autoantibody was diagnosed at 16 weeks. The median duration of diabetes at sampling was 7.9 months (IQR 2.2 months, 5.3 years). All individuals were treated with insulin from diagnosis and the median insulin dose at referral was 0.81 U/kg daily (IQR 0.50, 1.00). The median HbA_1c_ of these individuals was 65 mmol/mol (IQR 58, 78) (8.1% [IQR 7.5, 9.3]) and median blood glucose at diagnosis was 30 mmol/l (IQR 23, 38.1).

The number of individuals who had a first-degree relative (parent or sibling) with type 1 diabetes was 10/61 (16%), in keeping with rates reported in other studies of family history in type 1 diabetes [22]. The number of individuals who were the result of a consanguineous union (parents being second cousins or more closely related) was 15 (24%), similar to the rate in our entire cohort of individuals diagnosed at <6 months of age (332/1438 [23%]) and therefore likely reflecting the pattern of international referrals to our centre.

## Discussion

We have shown that type 1 diabetes can present before the age of 6 months using the combined measurement of a T1D-GRS, three islet autoantibodies and C-peptide. The combination of high levels of islet autoantibody positivity, reduced C-peptide and high type 1 diabetes genetic susceptibility makes a strong case that these individuals have polygenic type 1 diabetes. To our knowledge, type 1 diabetes is the first example of a polygenic autoimmune disease presenting before the age of 6 months. We identified individuals who were diagnosed in the first weeks of life and had low birthweight, suggesting reduced insulin secretion in utero and therefore reduced insulin-mediated growth. Our results therefore increase focus on how the nascent immune system might develop islet autoimmunity, and the circumstances that make type 1 diabetes possible at such young ages.

We identified individuals with type 1 diabetes below the age of 6 months. There was a marked excess of individuals with a T1D-GRS >95th centile of the normal population (63/166 vs 8/166 expected) in our cohort of 166 individuals diagnosed at <6 months of age who did not have a pathogenic variant in any of the 26 known genetic causes of NDM. By taking only the excess numbers, we conservatively estimate that the proportion of type 1 diabetes in our international cohort of 1438 cases of diabetes diagnosed before age 6 months would be 4%.

There are very few studies assessing type 1 diabetes under the age of 6 months. Huopio et al studied diabetes diagnosed at <12 months of age in Finland and showed that type 1 diabetes was more likely in individuals diagnosed at >6 months of age and monogenic diabetes was more likely in those diagnosed at <6 months of age [23]. However, some of the reported cases of diabetes diagnosed at <6 months of age were likely to be type 1 diabetes, as evidenced by 5/30 individuals not having a pathogenic variant in any of the genes tested and being islet autoantibody positive. Our study in 2016 analysed type 1 diabetes genetic risk and highlighted potential type 1 diabetes in individuals diagnosed before the age of 6 months, although we did not assess established biomarkers (islet autoantibodies and C-peptide) [7]. Another potential source of information is longitudinal birth studies but considering the incidence of all diabetes under the age of 6 months (1/20,000 to 1/500,000 live births) [24–28], unsurprisingly none of these have identified a case of type 1 diabetes diagnosed this early.

Autoantibodies to islet antigens are a hallmark feature of older children with type 1 diabetes, with >90% of individuals being positive for GADA, IA2A and/or ZnT8A autoantibodies [9]. We observed at least one islet autoantibody in 41% of those diagnosed with diabetes at <6 months of age with high T1D-GRS and in 58% of those diagnosed between the ages of 6 months and 2 years. The lower number of antibody-positive individuals diagnosed at <6 months of age with high T1D-GRS, when compared with individuals with older-onset type 1 diabetes, may be partially explained by IAA positivity. Eighty-two per cent of individuals diagnosed before the age of 6 months with high T1D-GRS were IAA positive compared with 39% of those with non-autoimmune monogenic NDM. However, we cannot rule out that this is due to immunity to exogenous insulin. We were not able to exclude maternal transfer of antibodies, although none of the individuals who were antibody positive had a mother with type 1 diabetes, and the cut-offs used to define positivity (99th centile of healthy individuals) mean that we would only expect 1% of the non-diabetic population to be positive for any antibody. A low number of individuals with monogenic NDM were positive for a single antibody, in keeping with the false-positive rate expected by the threshold used (*p* = 0.3 for all antibodies, *p* > 0.4 for each individual antibody). The T1D-GRS was similar in the individuals with monogenic diabetes cases who were positive vs negative for an antibody (median 0.236 vs 0.233, *p* = 0.7). No individuals with monogenic NDM were positive for >1 antibody.

A characteristic of type 1 diabetes diagnosed at <6 months of age was rapid beta cell destruction. We found that 14/19 (74%) of individuals diagnosed before the age of 6 months with high T1D-GRS had undetectable C-peptide (<3 pmol/l) when measured at follow-up. Only three of the seven individuals studied within a year of diagnosis (median 1 month) had detectable C-peptide and levels were all below 39 pmol/l. This is in keeping with previous data indicating that, in type 1 diabetes, earlier age of diagnosis is associated with rapid progression to severe insulin deficiency: children diagnosed before the age of 18 years have faster progression than those diagnosed as adults [29, 30] and children diagnosed before the age of 5 years show faster progression than those diagnosed after the age of 5 years [31]. Additionally, longitudinal studies have shown that children at risk of type 1 diabetes who develop islet autoantibodies early show faster progression to overt disease, supporting a more rapid onset and shorter presymptomatic phase in younger children [32]. The faster progression to insulin deficiency may represent similar rates of decline in beta cell mass but lower initial beta cell mass in those diagnosed at a young age [33, 34]. This may be particularly relevant to our cohort given that the expansion of beta cell mass in humans is limited to very early life [35]. Our study is cross sectional and longitudinal studies are needed to properly define C-peptide decline in type 1 diabetes diagnosed at <6 months of age.

An interesting and unique finding in our study is the low birthweight in our cohort of individuals diagnosed at <6 months of age with high T1D-GRS. This was most marked in those diagnosed in the first 3 months of life, with a reduction in corrected birthweight of approximately 900 g (SD −1.98). This raises the possibility that there is a common explanation for the low birthweight and extremely early onset of diabetes in these individuals. This is compatible with the severe insulin deficiency that results in a presentation of diabetes soon after birth existing in utero, resulting in reduced insulin-mediated growth and hence lower birthweight. Insulin is a potent fetal growth factor and absent fetal insulin secretion in individuals with pancreatic agenesis results in greatly reduced birthweight (approximately −1400 g, SD 3) [36, 37]. In immunodysregulation polyendocrinopathy enteropathy X-linked (IPEX) syndrome, a monogenic autoimmune disease caused by hemizygous pathogenic *FOXP3* variants [12], islet autoimmunity has been observed in pancreases from affected miscarried fetuses showing that it is possible to develop islet autoimmunity in utero [38]. Other possible explanations include maternal factors that could cause low birthweight such as unrecognised infection/illness or nutritional deficits during pregnancy [39]. Previous studies in type 1 diabetes after 6 months have shown a modest increase (HR 1.13) in overall risk of type 1 diabetes in large for gestational age babies [40, 41]. Individuals with a low T1D-GRS also showed reduced birthweights (*z* score −1.23), which may reflect as-yet unknown monogenic causes of NDM, where there is often a defect resulting in reduced or absent insulin secretion during fetal development. We did not see an association with low birthweight in children with type 1 diabetes diagnosed between the ages of 6 months and 2 years, suggesting that this is a specific feature of very-young-onset diabetes.

This is the first characterisation of polygenic autoimmunity diagnosed in the first 6 months of life. The neonatal immune system is immature and biased towards tolerance at birth. However, it undergoes rapid change in the first months of life to prepare infants for diverse immune challenges [42]. Primary defects in the immune system, particularly in regulatory T cell function, are known to cause autoimmunity including autoimmune diabetes in neonates [43]. This suggests that a major failure of early immune tolerance mechanisms may underlie type 1 diabetes diagnosed before the age of 6 months. This failure may be triggered by environmental factors such as specific viral infection during pregnancy or in the first few months of life [44]. Characterisation of microbiome, virome and immune phenotype in individuals diagnosed at <6 months of age with high T1D-GRS could provide mechanistic insights into how the nascent immune system can develop a specific targeted attack against beta cells.

Our study further demonstrates the utility of polygenic risk scores for disease stratification and studying rare subtypes. Unlike biomarkers, genetic information does not change over time and is easily tested using a small volume of blood or saliva. Furthermore, DNA should be readily available from all individuals diagnosed with diabetes before the age of 6 months as they are commonly referred for genetic testing of NDM genes [1].

While we have demonstrated that type 1 diabetes can present under the age of 6 months, comprehensive genetic testing is still essential for all infants diagnosed with diabetes below the age of 6 months as 85–90% have a known monogenic cause and in ~50% of those a genetic diagnosis can optimise therapy [45]. This testing is available free for any individual diagnosed at <6 months of age, whatever their current age (www.diabetesgenes.org). Monogenic diabetes is identified in approximately 5% of individuals diagnosed at 6–9 months of age and in this age group polygenic type 1 diabetes is more likely [15]. In this age group it is therefore important to assess all the clinical information available as genetic testing may be indicated in some individuals. Furthermore, as syndromic presentation is common in monogenic disease, individuals diagnosed with diabetes at >6 months of age with additional features (e.g. exocrine insufficiency, autoimmune disorders, liver disease, neurological features) or with other factors making a monogenic cause more likely (e.g. consanguinity or a family history suggesting mendelian inheritance) should be considered for genetic testing. It is important to note that these guidelines are pragmatic and not perfect; there are rare cases of monogenic diabetes diagnosed in the first years of life that remain difficult to identify using cut-off points and rely on the observation of astute clinicians. The T1D-GRS is a useful tool for the proportion of individuals in whom a genetic mutation has been ruled out by comprehensive testing. As such, it may be time and cost effective to include it on NDM genetic testing panels so that the data are available if no monogenic cause is identified. When a genetic cause is ruled out, islet autoantibody status may be useful in combination with the T1D-GRS. However, islet autoantibody positivity does not preclude a monogenic cause as individuals with monogenic autoimmunity (e.g. IPEX syndrome) commonly have islet autoantibodies and may present at >6 months of age. It is rare but possible for islet autoantibodies to appear after diagnosis in type 1 diabetes [46], therefore longitudinal study of islet autoantibodies in this very young group is warranted as it is possible that antibodies could appear later as the immune system matures.

Our study has some limitations. We were unable to unequivocally diagnose all infants as having type 1 diabetes at <6 months of age as a small proportion of individuals with monogenic diabetes have a high T1D-GRS (~5%) due to the distribution of risk alleles in the population. However, all individuals were screened and were negative for pathogenic variants in the 26 known genetic causes. A major limitation is that we were unable to measure autoantibodies or C-peptide in all individuals as we did not have sufficient samples for the analysis. Many samples for autoantibody measurement were collected years after diagnosis of diabetes and we are therefore unable to rule out loss or gain of islet autoantibodies during the intervening time. Our sample collection for C-peptide measurement was random, rather than being under controlled conditions. However, we found that individuals with type 1 diabetes (diagnosed at <6 months of age or between 6 months and 2 years of age) had lower C-peptide levels than those with monogenic NDM, supporting the validity of the results.

In conclusion, we have shown that type 1 diabetes can present before the age of 6 months and is characterised by rapid beta cell loss and islet autoimmunity. This extreme form of autoimmune diabetes is the first polygenic autoimmune disease demonstrated to present before the age of 6 months, challenging current understanding of the early immune system.

## Electronic supplementary material

ESM (PDF 384 kb)

## Data Availability

The genotype and clinical data in this study could be used to identify individuals and so cannot be made openly available. Access to data is open only through collaboration. Requests for collaboration will be considered following an application to the Genetic Beta Cell Research Bank (https://www.diabetesgenes.org/current-research/genetic-beta-cell-research-bank/). Contact by email should be directed to the Lead Nurse, Dr Bridget Knight (b.a.knight@exeter.ac.uk).

## Abbreviations

GADA: GAD autoantibody
IAA: Insulin autoantibody
IA2A: Insulinoma antigen-2 autoantibody
IPEX: Immunodysregulation polyendocrinopathy enteropathy X-linked
NDM: Neonatal diabetes
T1D-GRS: Type 1 diabetes genetic risk score
ZnT8A: Zinc transporter 8 autoantibody
