# Supplementary material for: Type 1 diabetes can present before the age of 6 months and is characterised by autoimmunity and rapid loss of beta cells
Source: Diabetologia. 2020 Oct 8;63(12):2605–15. doi: 10.1007/s00125-020-05276-4 (PMC7641942; doi:10.1007/s00125-020-05276-4)
Supplement: Supplementary file 1 — (PDF 384 kb) [file 125_2020_5276_MOESM1_ESM.pdf]

Electronic supplementary material:

# Type 1 Diabetes can present before the age of 6 months and is characterized by autoimmunity and rapid loss of beta-cells

Matthew B Johnson[1*], Kashyap A Patel[1*], Elisa De Franco[1], William Hagopian[2], Michael Killian[2], Timothy J McDonald[1,3], Timothy IM Tree[4,5], Clara Domingo-Vila[4], Michelle Hudson[1,6], Suzanne Hammersley[1,6], Rebecca Dobbs[1,6],EXE-T1D consortium[#], Sian Ellard[1], Sarah E Flanagan[1], Andrew T Hattersley[1], Richard A Oram[1]

*These authors contributed equally to this manuscript

1. Institute of Biomedical and Clinical Science, University of Exeter Medical School, UK
2. Pacific Northwest Research Institute, Seattle, WA., U.S.A.
3. Blood Sciences, Royal Devon & Exeter NHS Foundation Trust, Exeter, U.K.
4. Department of Immunobiology, School of Immunobiology & Microbial Sciences, Kings College London, London, U.K.
5. NIHR Biomedical Research Centre Guys and St Thomas' NHS Foundation Trust and Kings College London, London, U.K.
6. National Institute for Health Exeter Research Clinical Research Facility, Royal Devon and Exeter NHS Foundation Trust, Exeter, U.K.

**EXE-T1D consortium**

Luke Weymouth[1], Cate Speake[2], Michael N Weedon[3], Bart O Roep[4]

1. Exeter National Institute for Health Research Clinical Research Facility, Royal Devon and Exeter NHS Foundation Trust, Exeter, U.K.
2. Diabetes Clinical Research Program, Benaroya Research Institute at Virginia Mason, Seattle, Washington.
3. Institute of Biomedical and Clinical Science, University of Exeter Medical School, UK
4. Department of Diabetes Immunology, Diabetes and Metabolism Research Institute, Beckman Research Institute of City of Hope, Duarte, CA, USA.

**ESM table 1:**

| Gene/locus | Inheritance |
|---|---|
| *ABCC8* | Autosomal dominant/recessive |
| *CNOT1* | Autosomal dominant |
| *EIF2AK3* | Autosomal recessive |
| *FOXP3* | X-linked recessive |
| *GATA4* | Autosomal dominant |
| *GATA6* | Autosomal dominant |
| *GCK* | Autosomal recessive |
| *GLIS3* | Autosomal recessive |
| *HNF1B* | Autosomal dominant |
| *IER3IP1* | Autosomal recessive |
| *IL2RA* | Autosomal recessive |
| *INS* | Autosomal dominant/recessive |
| *KCNJ11* | Autosomal dominant |
| *LRBA* | Autosomal recessive |
| *MNX1* | Autosomal recessive |
| *NEUROD1* | Autosomal recessive |
| *NEUROG3* | Autosomal recessive |
| *NKX2.2* | Autosomal recessive |
| *PDX1* | Autosomal recessive |
| *PTF1A* | Autosomal recessive |
| *RFX6* | Autosomal recessive |
| *SLC19A2* | Autosomal recessive |
| *SLC2A2* | Autosomal recessive |
| *STAT3* | Autosomal dominant |
| Trisomy 21 | Aneuploidy |
| *WFS1* | Autosomal dominant/recessive |

The 26 known genetic causes of neonatal diabetes tested in all patients diagnosed with diabetes <9 months.

**ESM table 2:**

| ***SNP ID*** | ***Gene*** | ***Odds Ratio*** | ***Weight*** |
|---|---|---|---|
| rs2187668, rs7454108 | *DR3/DR4-DQ8* | *48.18* | *3.87* |
| | *DR3/DR3* | *21.12* | *3.05* |
| | *DR4-DQ8/DR4-DQ8* | *21.98* | *3.09* |
| | *DR4-DQ8/X* | *7.03* | *1.95* |
| | *DR3/X* | *4.53* | *1.51* |
| rs1264813 | *HLA_A_24* | *1.54* | *0.43* |
| rs2395029 | *HLA_B_5701* | *2.5* | *0.92* |
| rs3129889 | *HLA_DRB1_15* | *14.88* | *2.70* |
| rs2476601 | *PTPN22* | *1.96* | *0.67* |
| rs689 | *INS* | *1.75* | *0.56* |
| rs12722495 | *IL2RA* | *1.58* | *0.46* |
| rs2292239 | *ERBB3* | *1.35* | *0.30* |
| rs10509540 | *C10orf59* | *1.33* | *0.29* |
| rs4948088 | *COBL* | *1.30* | *0.26* |
| rs7202877 | | *1.28* | *0.25* |
| rs12708716 | *CLEC16A* | *1.23* | *0.21* |
| rs3087243 | *CTLA4* | *1.22* | *0.20* |
| rs1893217 | *PTPN2* | *1.20* | *0.18* |
| rs11594656 | *IL2RA* | *1.19* | *0.17* |
| rs3024505 | *IL10* | *1.19* | *0.17* |
| rs9388489 | *C6orf173* | *1.17* | *0.16* |
| rs1465788 | | *1.16* | *0.15* |
| rs1990760 | *IFIH1* | *1.16* | *0.15* |
| rs3825932 | *CTSH* | *1.16* | *0.15* |
| rs425105 | | *1.16* | *0.15* |
| rs763361 | *CD226* | *1.16* | *0.15* |
| rs4788084 | *IL27* | *1.16* | *0.15* |
| rs17574546 | | *1.14* | *0.13* |
| rs11755527 | *BACH2* | *1.13* | *0.12* |
| rs3788013 | *UBASH3A* | *1.13* | *0.12* |
| rs2069762 | *IL2* | *1.12* | *0.11* |
| rs2281808 | | *1.11* | *0.1* |
| rs5753037 | | *1.1* | *0.1* |

Single Nucleotide Polymorphisms used to generate the type 1 diabetes genetic risk score.

**ESM table 3:**

| Feature | Islet autoantibody tested (n=22) | Islet autoantibody unavailable (n=41) | p value |
|---|---|---|---|
| **T1D-GRS** | 0.297 (0.292, 0.305) | 0.296 (0.292, 0.305) | 0.7 |
| **Related parents** | 6/22 (27%) | 9/41 (22%) | 0.8 |
| **Birthweight Z score** | -0.71 (-1.97, -0.03) [n=20] | -1.23 (-1.89, -0.09) [n=28] | 0.7 |
| **Female** | 9 (41%) | 18 (44%) | 1.0 |
| **First degree relative with type 1 diabetes** | 3/22 (14%) | 7/39 (18%) | 0.7 |
| **Age diagnosed (weeks)** | 16 (6, 21) | 19 (8, 22) | 0.6 |
| **Duration diabetes (years)** | 3.0 (0.2, 7.5) | 0.5 (0.2, 1.7) | 0.2 |
| **Syndromic presentation** | 3 (14%) | 3 (7%) | 0.4 |
| **Additional autoimmune disease** | 0 (0%) | 3 (7%) | 0.5 |

Comparison of characteristics of those where islet autoantibody testing was possible vs. not possible in individuals with diabetes diagnosed <6 months and a high T1D-GRS.

**ESM table 4:**

| Feature | T1D <3m (n=24) | T1D 3-6m (n=39) | P |
|---|---|---|---|
| Age at diagnosis | 3w (2d, 8w) | 20 (17, 24) | <0.0001 |
| Birthweight Z-score | -1.98 (-2.39, -1.23) | -0.53 (-1.24, 0.13) | 0.0001 |
| T1D-GRS | 0.299 (0.293, 0.307) | 0.296 (0.291, 0.303) | 0.3 |
| Female | 13/24 (54%) | 14/39 (36%) | 0.2 |
| IAA positive | 3/7 (43%) | 15/15 (100%) | 0.005 |
| GAD/IA2/ZnT8 positive | 0/7 (0%) | 9/15 (60%) | 0.02 |
| GAD positive | 0 | 6/15 (40%) | 0.1 |
| IA2 positive | 0 | 4/15 (24%) | 0.1 |
| ZnT8 positive | 0 | 3/15 (20%) | 0.3 |
| Serum C-peptide | 2.9 (2.9, 10) [n=7] | 2.9 (2.9, 2.9) [n=12] | 0.4 |
| Duration diabetes at testing | 4m (5w, 3y) | 1y (10w, 5.5y) | 0.3 |

Comparison of patients with a high T1D-GRS and no known cause with a diagnosis <3 months vs 3-6 months.

**ESM figure 1:**

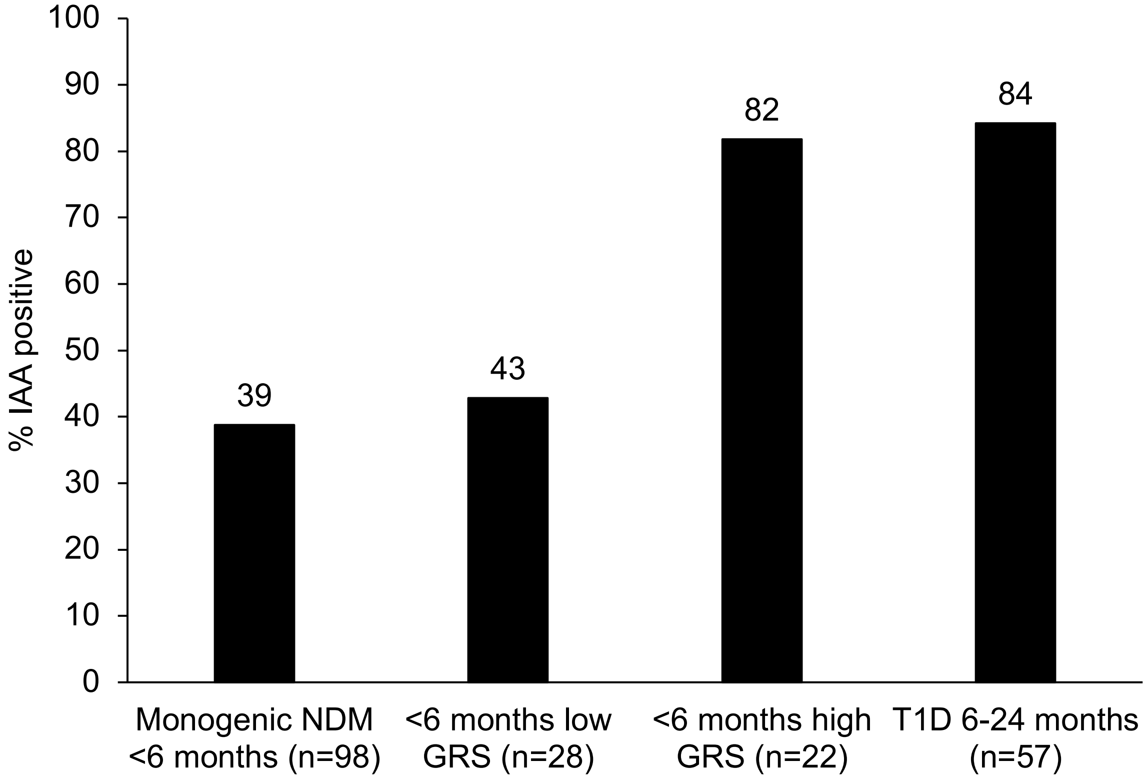


IAA positivity in monogenic neonatal diabetes, diabetes diagnosed <6 months without a known cause and a low GRS, diabetes diagnosed <6 months without a known cause and a high GRS, and type 1 diabetes diagnosed 6-24 months. All patients were insulin treated for >2 weeks at testing.

**ESM figure 2:**

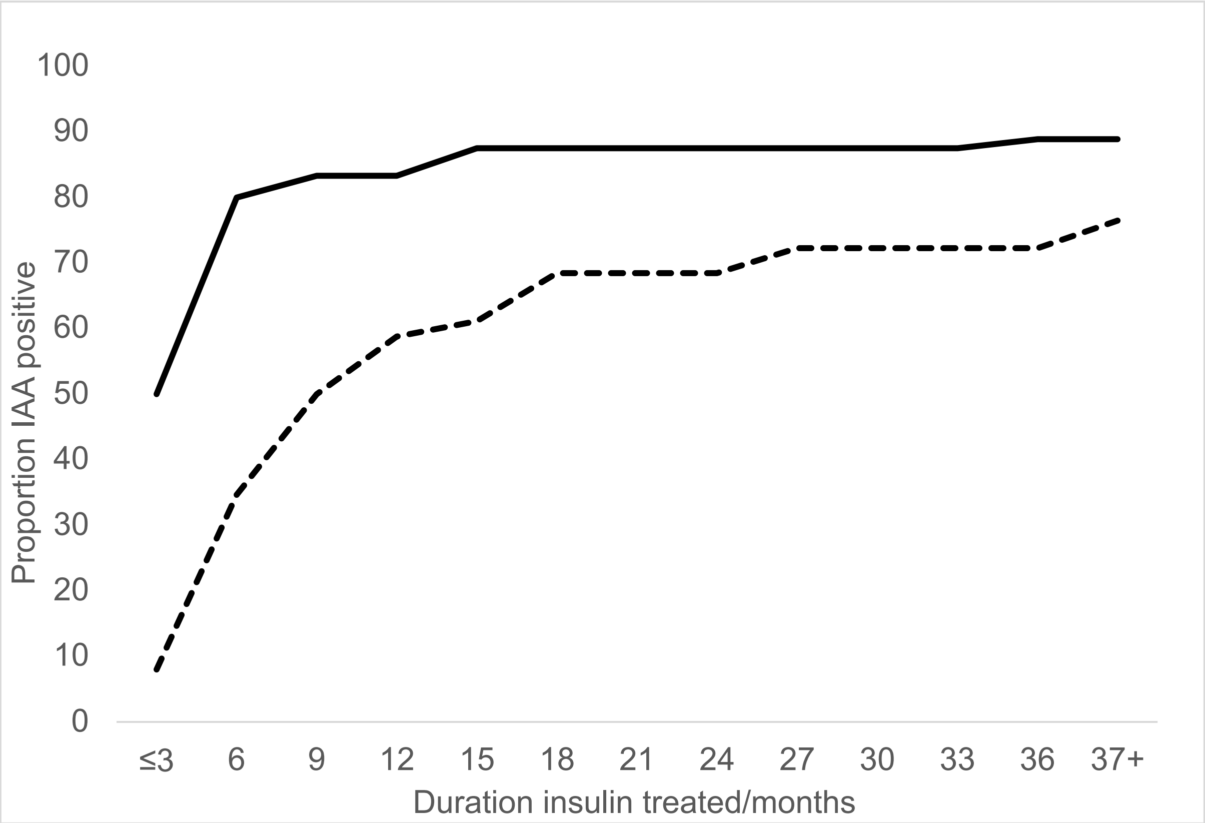


Proportion of individuals positive for insulin autoantibody (IAA) at different durations of insulin treatment. Black line = diabetes diagnosed <6 months without a known cause and high GRS. Dashed line = confirmed monogenic neonatal diabetes. Where patients were positive for an antibody at testing, they were assumed to remain positive. Where patients were negative at testing, they were assumed to have always been negative but censored at testing.

**ESM figure 3:**

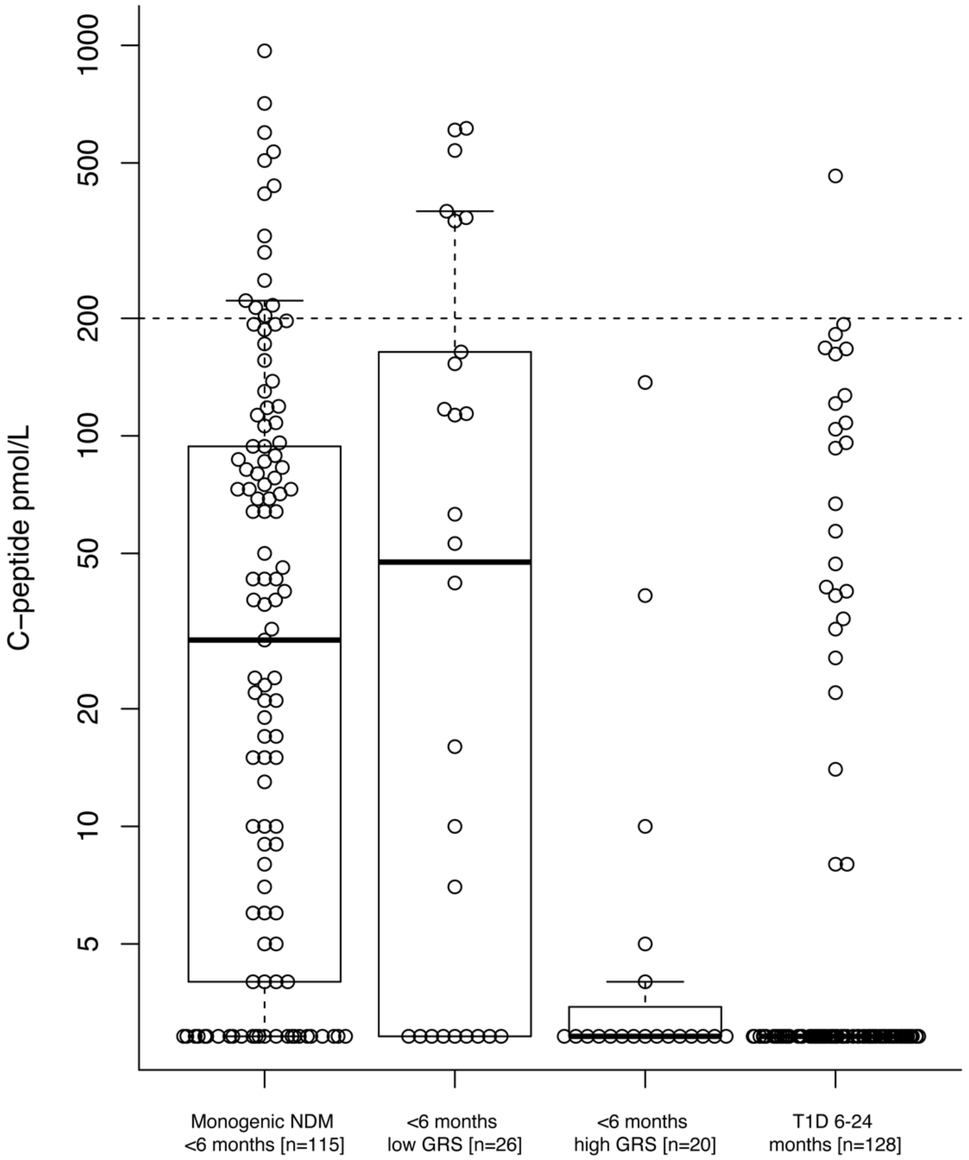


Serum C-peptide (pmol/L) in controls with a known monogenic cause, unknown diabetes diagnosed <6 months with a low T1D-GRS, unknown diabetes diagnosed <6 months with high T1D-GRS and type 1 diabetes diagnosed between 6 and 24 months. C-peptide is plotted on a log scale. The dashed horizontal line represents 200pmol/L. Median duration was 1 year (range 2 days – 20 years).

**ESM figure 4:**

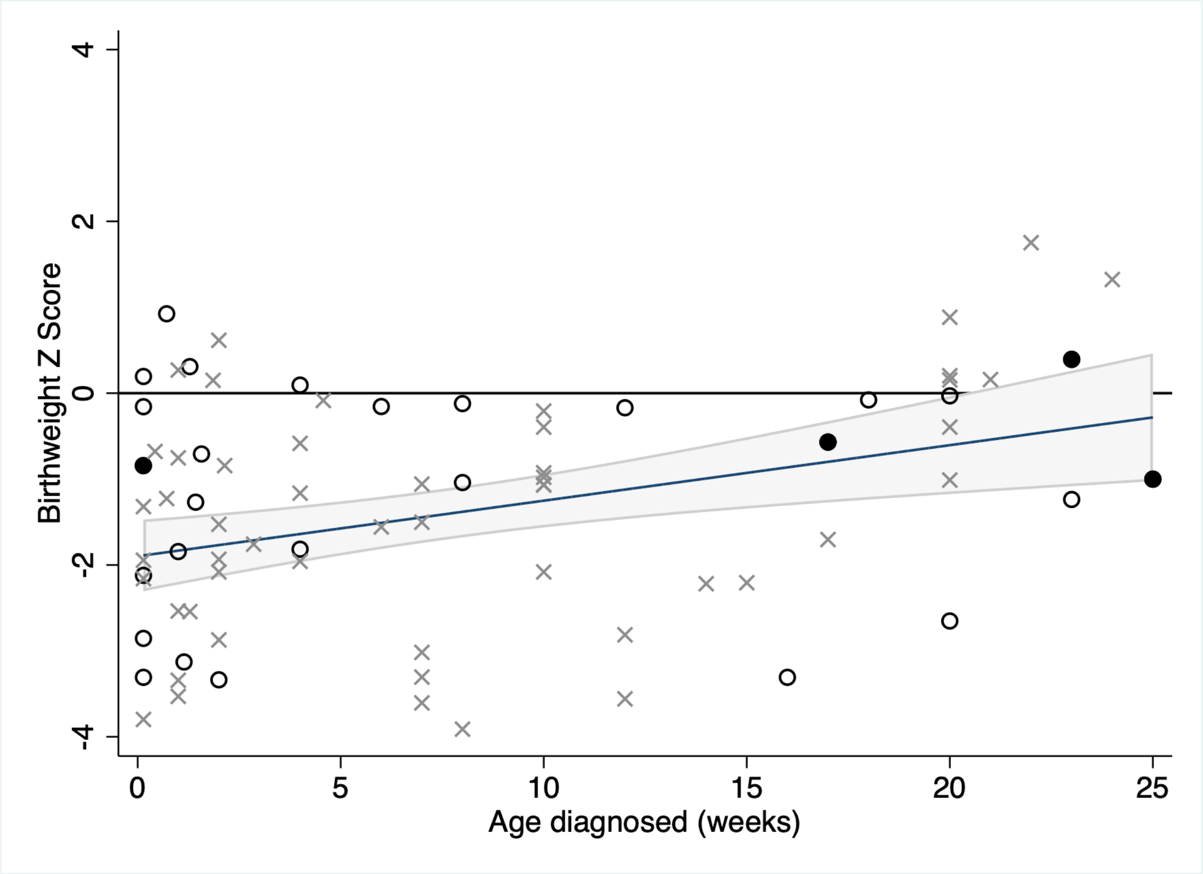


Scatter plot of adjusted birth weight Z-score and age at diagnosis in weeks individuals without a known monogenic cause with low T1D-GRS (n=81). The fitted line is the predicted linear regression ($r^2$=0.11, $P$=0.001) with grey shading representing the 95% confidence interval. Filled black dots – islet autoantibody positive. Hollow dots – islet autoantibody negative. Grey crosses – islet autoantibody testing unavailable.
